# Biomarkers for intensive care unit-acquired weakness: a systematic review for prediction, diagnosis and prognosis

**DOI:** 10.1186/s13613-025-01500-9

**Published:** 2025-07-02

**Authors:** Jiamei Song, Ting Deng, Qingmei Yu, Xun Luo, Yanmei Miao, Leiyu Xie, Yongming Mei, Peng Xie, Shaolin Chen

**Affiliations:** 1 Nursing Department, Affiliated Hospital of Zunyi Medical University, No. 149, Dalian Road, Huichuan District, Zunyi City, 563000 Guizhou Province China; 2 School of Nursing, Zunyi Medical University, No.6, Xuefu West Road, Xinpu New District, Zunyi City, 563000 Guizhou Province China; 3 Department of Critical Care Medicine, Affiliated Hospital of Zunyi Medical University, No. 149, Dalian Road, Huichuan District, Zunyi, 563000 Guizhou Province China; 4 Department of Critical Care Medicine, the Third Affiliated Hospital of Zunyi Medical University (The First People’s Hospital of Zunyi City), Feng Huang Road 98, Huichuan District, Zunyi, 563000 Guizhou Province China; 5 Public Experimental Center of Clinical Medicine, Affiliated Hospital of Zunyi Medical University, No.149, Dalian Road, Huichuan District, Zunyi City, 563000 Guizhou Province China; 6 Department of Critical Care Medicine, Hengyang Medical School, University of South China, Hengyang, 421200 Hunan Province China

**Keywords:** ICU-acquired weakness, Biomarkers, Diagnosis, Prognosis, Prediction, Systematic review

## Abstract

**Background:**

Intensive care unit-acquired weakness (ICU-AW) is a common and debilitating complication in critically ill patients, significantly affecting both short- and long-term outcomes. The existing ICU-AW diagnostic methods are not widely accepted and have a narrow application window. Biomarkers offer potential for diagnosing, predicting, and prognosticating ICU-AW, but a comprehensive synthesis of the available evidence is still lacking.

**Methods:**

We conducted a systematic search across PubMed, Cochrane Library, Embase, Web of Science, CNKI, Wanfang Database, China Science and Technology Journal Database (VIP Database), and China Biomedical Literature Database (SinoMed Database) from inception to January 23, 2025. Study quality was assessed using the revised Newcastle-Ottawa scale and the Quality Assessment of Diagnostic Accuracy Studies-2 tool. Data extraction included basic characteristics of the included studies, name of biomarkers, objective, specimen types, sampling time, type of biomarker, ICU-AW diagnostic criteria, and outcomes.

**Results:**

Out of 5,769 publications screened, 11 studies of moderate to high quality (scores ≥ 6) involving 1,176 critically ill patients were included. Ten biomarkers were identified and categorized into five mechanisms: muscle injury (myoglobin, N-titin, urinary titin), metabolic pathway (glucose transporter protein type-4), neurological injury (neurofilament light/heavy chain), stress response (growth differentiation factor-15), and inflammatory process (monocyte chemoattractant protein-1, NETs marker cfDNA, and miR-181a). Six biomarkers demonstrated strong predictive and diagnostic accuracy with AUC values exceeding 0.80. Notably, growth differentiation factor-15 exhibited excellent clinical utility across diagnostic, predictive, and prognostic applications (AUC ≥ 0.85). The remaining four biomarkers showed moderate performance, with AUC values ranging from 0.60 to 0.80.

**Conclusion:**

While ten biomarkers exhibit potential for ICU-AW assessment, their clinical utility remains inconsistent. This highlights the need for large-scale, prospective validation studies and the incorporation of advanced technologies to refine existing biomarkers and identify novel candidates for ICU-AW prediction, diagnosis and management.

**Date of registration:**

Registered 1 August 2024.

**Trial registration:**

PROSPERO ID: CRD42024574437.

**Supplementary Information:**

The online version contains supplementary material available at 10.1186/s13613-025-01500-9.

## Introduction

Intensive care unit-acquired weakness (ICU-AW) is a common and severe complication in critically ill patients, encompassing critical illness polyneuropathy (CIP), critical illness myopathy (CIM) and critical illness neuromyopathy (CINM) [1]. It manifests as neuromuscular dysfunction affecting both limbs and respiratory muscles [2], often resulting from multifactors like severe sepsis, prolonged immobilization, and mechanical ventilation [3]. ICU-AW significantly increases the risks of prolonged mechanical ventilation, ventilator-associated complications, higher mortality, and long-term disability [4]. Therefore, early prediction and identification are extremely crucial for timely interventions to prevent or mitigate adverse outcomes and improve patient outcomes [5].

However, current diagnostic methods have notable limitations [6]. The Medical Research Council (MRC) score, though quick, is subjective, possessed a ceiling effect and limited to conscious patients [7]. Neuroelectrophysiological tests, such as electromyography, provide detailed insights but are invasive, require specialized expertise, and lack standardization [8–10]. Muscle ultrasound is non-invasive but can be affected by edema, obesity, and operator variability [11]. Muscle biopsy provides definitive diagnosis but is invasive, costly, and complex, limiting routine use [2]. Biomarkers, defined as objective, quantifiable indicators of physiological or pathological processes, hold significant potential for improving the diagnosis, monitoring, and prognosis of various diseases [12]. Therefore, there is a critical need for non-invasive, simple, and objective biomarkers to enhance the early detection and management of ICU-AW [13, 14].

Several studies have explored biomarkers for predicting and identifying ICU-AW. Promising biomarkers, such as growth differentiation factor-15 (GDF-15), have excellent performance in predicting and diagnosing ICU-AW, and can also predict a 90 days survival rate [15, 16]. Myoglobin and monocyte chemoattractant protein-1 (MCP-1) have high predictive and diagnostic value, respectively [17, 18], while elevated microRNA-181a (miR-181a) during early ICU admission shows high specificity for predicting 7-day muscle atrophy in critically ill patients [19]. Peak levels of neurofilament light (NfL) and neurofilament heavy (NfH) chain exhibit good diagnostic and predictive accuracy for muscle weakness [20, 21]. Neutrophil Extracellular Traps (NETs) marker cell free DNA (cfDNA) and glucose transporter type-4 (GLUT-4), show predictive potential but are limited to specific populations [22, 23]. Additionally, N-titin and urinary titin are unsuitable for anuric patients, requiring further clinical validation [24, 25]. ICU-AW biomarkers face significant challenges, including limited validation across diverse populations and poor integration into clinical practice, hindering their routine use and underscoring a critical gap in current intensive care management.

The aim of this systematic review is to classify ICU-AW biomarkers based on their pathophysiological mechanisms, evaluate their validity and clinical applicability, and provide evidence to guide future research and enhance the translation of biomarker science into clinical practice for ICU-AW management. The following research questions are addressed in this review:

- What performance tested biomarkers related to ICU-AW have been investigated?
- What are the mechanisms underlying these biomarkers?
- What are their diagnostic, predictive and prognostic performance?

## Methods

Due to the importance of assessing research quality and the validation of biomarkers for clinical application, we chose a systematic review over a scoping review. Furthermore, the significant heterogeneity among the included studies made meta-analysis unfeasible. Our study adhered to the Preferred Reporting Items for Systematic Reviews and Meta-Analyses (PRISMA) guidelines [26], and was registered in the PROSPERO database (International Prospective Register of Systematic Reviews, registration number: CRD42024574437). Two researchers (SJM and DT) independently conducted the literature search, study screening, inclusion, quality and bias assessments, data extraction, synthesis, and analysis. Any disagreements were resolved through discussion or consultation with the corresponding author (CSL). Multiple rounds of team discussions were held to ensure the accuracy of study inclusion, data extraction, synthesis, and analysis, thereby enhancing the rigor and comprehensiveness of evidence synthesis for the multifactorial condition of ICU-AW.

### Study eligibility criteria

Studies were included in this review if they met the following criteria: (1) Participants: Critically ill patients diagnosed with ICU-AW or any of its three subcategories (CIM, CIP, and CINM), or those diagnosed with Post-ICU syndrome exhibiting muscle weakness or atrophy; (2) Outcomes: Studies reporting performance indicators for biomarker-based diagnosis, prediction or prognosis, such as area under curve (AUC); (3) Study design: Interventional or observational studies; (4) Language: Publications available in Chinese or English.

The exclusion criteria were: (1) Animal and in vitro studies; (2) Non-research articles, including reviews, conference abstracts, books, case reports and methodological articles; (3) Duplicate publications; (4) Studies with inadequate data on baseline characteristics or outcomes; (5) Unavailable full-text articles.

### Search strategy and study selection

The search strategy was developed with the assistance of a library specialist to ensure all the potential studies. CNKI, Wanfang Database, China Science and Technology Journal Database (VIP Database), China Biomedical Literature Database (SinoMed Database), PubMed, Cochrane Library, Emabse and Web of Science were searched spanning from inception until January 23, 2025. References from the included studies, guidelines, and relevant reviews were manually screened to identify additional eligible studies. All identified publications were collected in EndNote. Two reviewers, independently screened titles and abstracts to identify potentially included studies. Subsequently, the full texts of potentially eligible studies were carefully examined on the basis of the eligibility criteria to determine inclusion. The search strategies and outcomes for the eight databases are detailed in Supplementary Appendix Table S1.

### Risk of bias assessment

Cohort and case-control studies were assessed using the Newcastle-Ottawa Scale (NOS) (0–9 points), categorized as high (7–9), moderate (4–6), or poor quality (0–3) [27]. Cross-sectional studies were evaluated with the 11-item Agency for Healthcare Research and Quality scale, scoring 1 point for “yes” and 0 for “no”/ “unclear”, with total scores classified as low (0–3), medium (4–7), or high quality (8–11) [28]. Randomized controlled trials was assessed using the revised Cochrane Risk of Bias Tool version 2.0 (RoB 2.0) [29]. For non-randomized controlled trials, the Joanna Briggs Institute critical appraisal tool was employed [30]. Diagnostic studies were evaluated by the Quality Assessment of Diagnostic Accuracy Studies tool-2 (QUADAS-2) in RevMan [31].

### Data extraction

Data extraction was performed independently by two researchers using a standardised data extraction form. The extracted data included the author’s name, publication year, biomarker names, objective, sampling time point, specimens, type of biomarker, test method of biomarker, diagnostic time point of ICU-AW, ICU-AW diagnostic criteria and main results such as AUC, 95% confidence interval (CI), cut-off value, specificity, sensitivity and other relevant data.

### Data synthesis and analysis

A narrative synthesis approach was used to integrate the descriptions and findings of the included studies, exploring the relationship between various biomarkers and ICU-AW. Given the significant heterogeneity in parameters, including target population, study design, quality and outcomes, meta-analysis was not feasible for this review.

## Results

### Search results and study selection

**Fig. 1:**
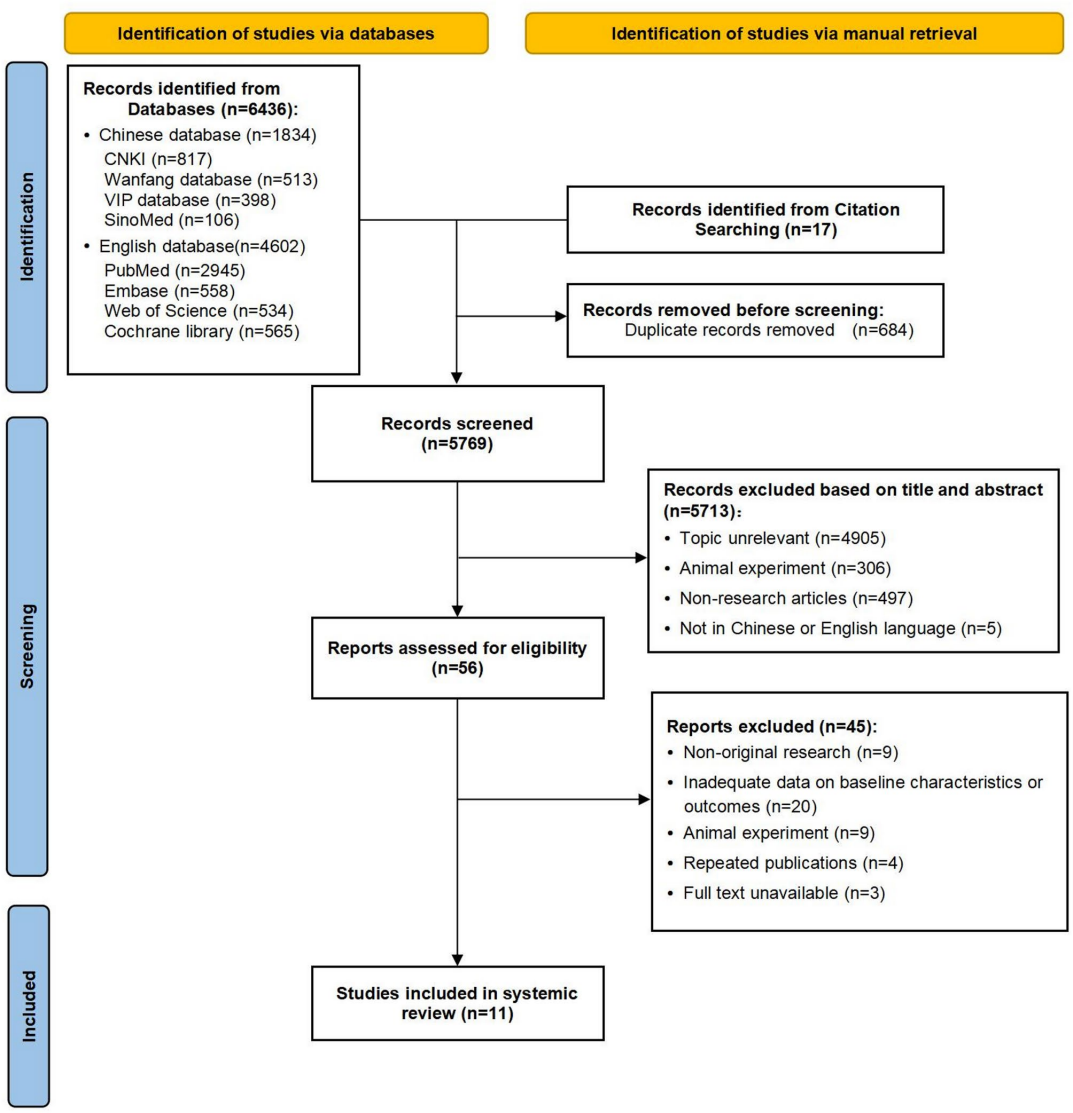
PRISMA 2020 flow diagram for study selection

A total of 6436 articles were initially retrieved from electronic databases, and additional 17 articles were manually searched. Following a comprehensive screening process, 11 cohort studies were included in this review. The selection process is presented in the flowchart (Fig. 1).

### Characteristics of the included studies

The 11 included studies were conducted across four countries: China (*n* = 6) [15–18, 21, 23], Japan (*n* = 2) [24, 25], the United Kingdom (*n* = 1) [19], and the Netherlands (*n* = 2) [20, 22]. Four studies were published in Chinese and seven in English. All studies employed a prospective design. All literature was published after 2014, and nine were published within the last five years. A total of 1,176 critically ill patients were included in this review, with sample sizes for each study ranging from 42 to 308 participants. Amongst these, 728 participants (61.9%) were male, and the mean or median age of participants ranged from 40 to 85 years. Table 1 outlines the characteristics of the included studies.

**Table 1:** Basic characteristics of the included studies

Study	Country	Study design	Type of study	Study population	Sample size	Age (years)	Gender (M/F)
						[M(QL, QU)]/χ̅ ± s	
Wieske 2014 [20]	Netherlands	Cohort study	Prospective study	Patients in ICU	ICU-AW: 18 Non-ICU-AW: 59	ICU-AW: 64 ± 15 Non-ICU-AW: 60 ± 15	ICU-AW: 10/8 Non-ICU-AW: 38/21
Bloch 2015 [19]	UK	Cohort study	Prospective study	Patients undergoing a high risk who require post-operative admission to adult critical care	ICU-AW: 23 Non-ICU-AW: 19	ICU-AW: 62.0 ± 16.2 Non-ICU-AW: 65.7 ± 17.2	ICU-AW: 12/11 Non-ICU-AW: 9/10
Xie 2020 [15]	China	Cohort study	Prospective study	Patients with acute respiratory failure, who received invasive mechanical ventilation in the ICU	ICU-AW: 50 Non-ICU-AW: 45	ICU-AW: 58.8 ± 13.6 Non-ICU-AW: 60.3 ± 15.6	ICU-AW: 32/18 Non-ICU-AW: 29/16
Nakano 2021 [24]	Japan	Cohort study	Prospective study	Patients admitted to the ICU who were expected to be hospitalised for more than 10 days	Low N-Titin/Cre Group: 25 High N-Titin/Cre Group: 25	Low N-Titin/Cre Group: 66.7 ± 14.9 High N-Titin/Cre Group: 75.4 ± 13.3	Low N-Titin/Cre Group: 17/8 High N-Titin/Cre Group:20/5
Wang 2022 [23]	China	Cohort study	Prospective study	Liver transplant recipients in ICU	ICU-AW: 17 Non-ICU-AW: 45	ICU-AW: 46 (40, 53) Non-ICU-AW: 46 (41, 50)	ICU-AW: 10/7 Non-ICU-AW: 31/14
Ding 2022 [18]	China	Cohort study	Prospective study	Patients with sepsis in ICU	ICU-AW: 31 Non-ICU-AW: 68	ICU-AW: 69.0 (52.0, 79.0) Non-ICU-AW: 58.5 (46.0, 68.8)	ICU-AW: 15/16 Non-ICU-AW: 44/24
Wang 2024 [17]	China	Cohort study	Prospective study	Patients with sepsis in ICU	ICU-AW: 129 Non-ICU-AW: 82	ICU-AW: 65.2 ± 18.9 Non-ICU-AW: 59.1 ± 17.0	ICU-AW: 84/45 Non-ICU-AW: 46/36
Huckriede 2021 [22]	Netherlands	Cohort study	Prospective study	ICU patients diagnosed as COVID-19	ICU Covid-19: 100 ICU Control: 11	ICU Covid-19: 62 (51–73) ICU Control: 70 (59–75)	ICU Covid-19: 74/26 ICU Control: 5/6
Zhao 2023 [21]	China	Cohort study	Prospective study	Patients with sepsis in ICU	ICU-AW: 24 Non-ICU-AW:41	ICU-AW: 65.50(48.00,78.25) Non-ICU-AW: 57.90(39.00,72.25)	ICU-AW: 10/14 Non-ICU-AW: 27/14
Guo 2023 [16]	China	Cohort study	Prospective study	Septicemia patients receiving mechanical ventilation	ICU-AW: 96 Non-ICU-AW: 212	ICU-AW: 68. 04 ± 7. 87 Non-ICU-AW: 65. 08 ± 9. 58	ICU-AW: 51/45 Non-ICU-AW: 131/81
Nakanishi 2020 [25]	Japan	Cohort study	Prospective study	Nonsurgical adult patients who were expected to remain in ICU ≥ 5 days	56	72 ± 13	33/23

Note: UK: The United Kingdom; ICU: intensive care unit; ICU-AW: intensive care unit-acquired weakness

### Quality of included studies

**Fig. 2:**
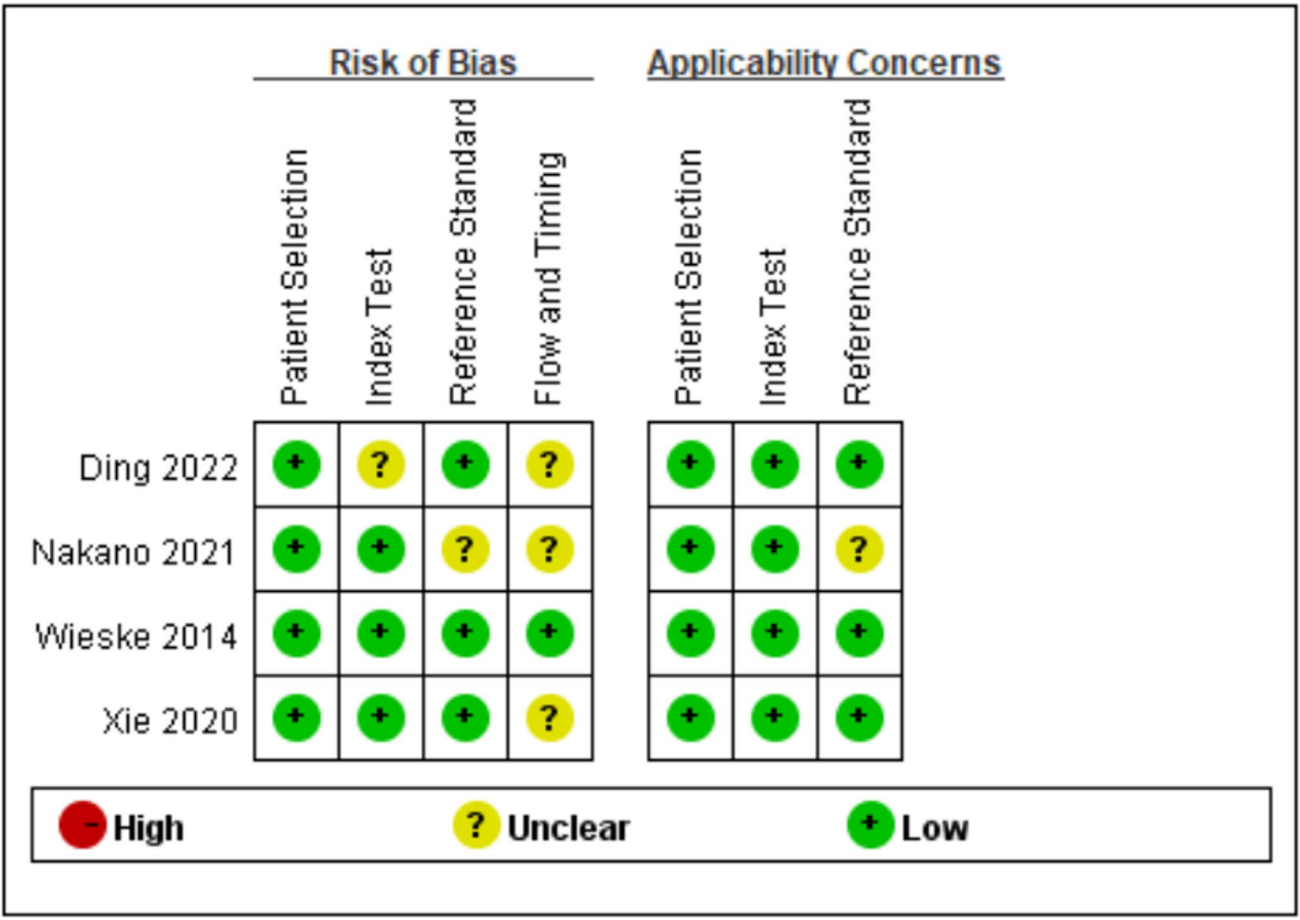
QUADAS-2 results of four diagnostic studies

Seven studies were rated as high quality based on NOS scores, and four as moderate quality (Supplementary Table S2), primarily due to inadequate assessment of result accuracy and bias. Figure 2 summarizes the QUADAS-2 evaluation for diagnostic studies, showing a generally low or unclear risk of bias [15, 18, 20, 24], mainly due to the lack of independent explanations for predefined thresholds or standard independent interpretation. The complete enter results of each study are detailed in Supplementary Table S3.

### Main outcomes of biomarkers associated with ICU-AW

This systematic review identified ten biomarkers associated with ICU-AW, categorized by their underlying mechanisms. GDF-15 was associated with stress response mechanisms [15, 16], while myoglobin, N-titin and urinary titin were linked to muscle injury [17, 24, 25]. MCP-1, NETs marker cfDNA and miR-181a were related to inflammatory processes [18, 19, 22]. NfL and NfH was closely associated with neurological injury [20, 21], and GLUT-4 played a role in the metabolic pathway of ICU-AW [23].

Of the studies included, nine collected blood samples [15–23], and two utilized urine sample [24, 25]. Biomarkers were predominantly detected using enzyme-linked immunosorbent assay (ELISA) in eight studies [15, 16, 18, 20, 21, 23–25], while others used quantitative-real time polymerase chain reaction [19, 22], and chemiluminescent assays [17]. The MRC scale was used to diagnose ICU-AW in ten studies [15–18, 20–25], and one studies used ultrasound [19]. Notably, six studies conducted continuous biomarker sampling at multiple time points [15, 18–20, 24, 25], while others collected single samples within 24 h of ICU admission [16, 17, 21–23].

**Fig. 3:**
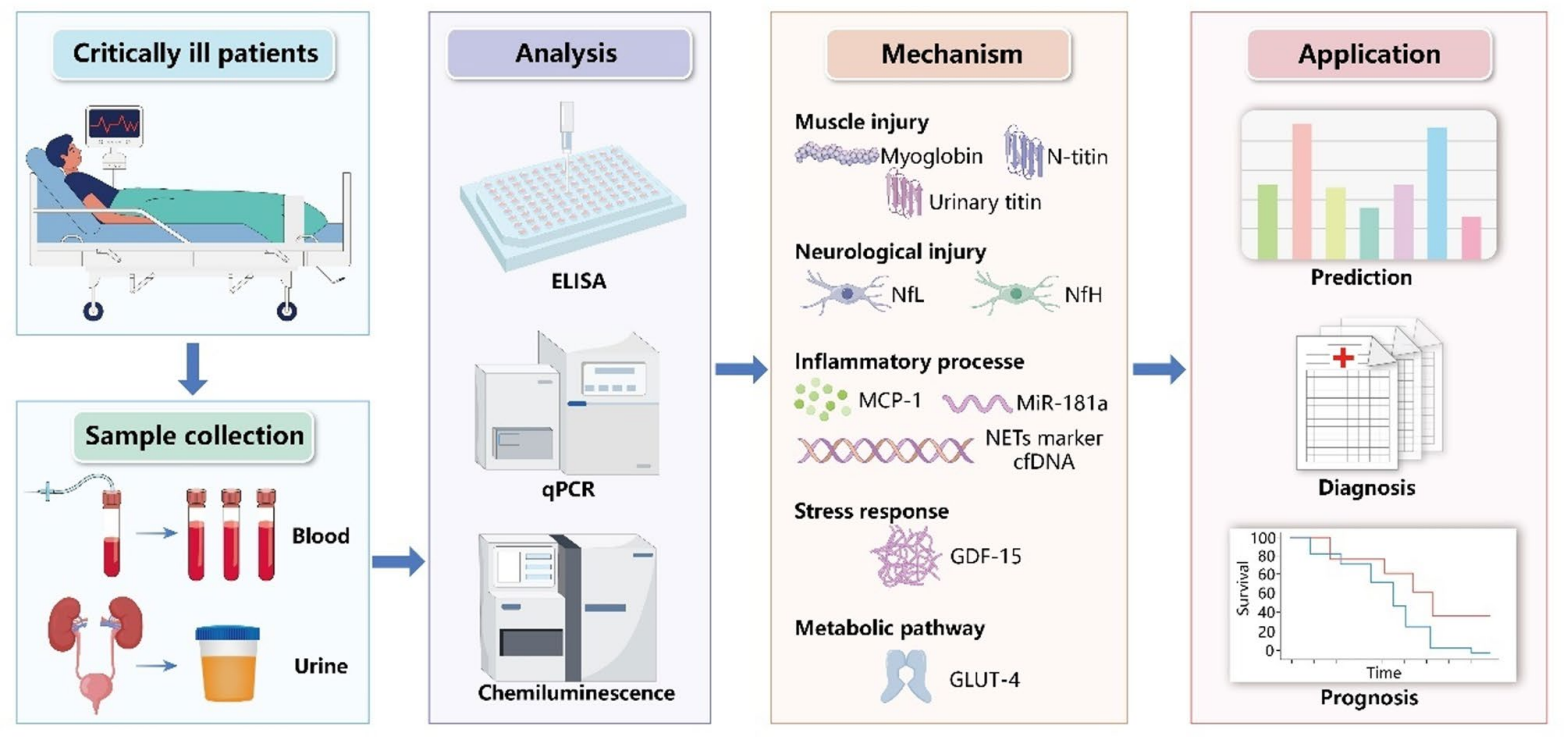
Analysis, mechanism, and application of ICU-AW biomarkers ELISA: enzyme linked immunosorbent assay; qPCR: Quantitative Real-time polymerase chain reaction; NfL: neurofilament light chain; NfH: neurofilament heavy chain; MCP-1: monocyte chemoattractant protein-1; miR-181a: microRNA-181a; NETs: neutrophil extracellular traps; cfDNA: cell-free DNA; GDF-15: growth differentiation factor-15; GLUT-4: glucose transporter type-4

The primary focus of most studies was to predict ICU-AW, accounting for 45.5% of the included research [16, 17, 19, 21, 23]. Three studies focused on diagnostic analyses [18, 20, 24]. One combined diagnostic and prognostic analyses [15], and two involved predictive and prognostic analyses [22, 25]. All studies reported AUC values for the biomarkers, ranging from 0.600 to 0.904, with sensitivity between 0.560 and 0.940, and specificity ranging from 0.544 to 0.961. Among the evaluated biomarkers, GDF-15, MCP-1, NfH, and N-titin demonstrated robust diagnostic performance with AUC values consistently exceeding 0.80. In the predictive biomarker category, only GDF-15, GLUT-4 and myoglobin showed comparable discriminative ability (AUC > 0.80), while urinary titin, NETs marker cfDNA, and miR-181a exhibited limited predictive value with AUC values ranging from 0.60 to 0.80. Notably, GDF-15 demonstrated a strong correlation with 90-day survival in mechanically ventilated ICU patients [15], while cumulative urinary titin level and NETs marker cfDNA was associated with ICU mortality [22, 25]. Detailed characteristics of the biomarkers related to ICU-AW are provided in Table 2; Fig. 3.

**Table 2:** Detailed characteristics of biomarkers associated with ICU-AW

Name of biomarker	Objective	Samples	Sampling time point	Type of biomarker	Diagnostic time point of ICU-AW	ICU-AW diagnostic criteria	Main results				Other
							AUC (95% CI)	Cut-off value	Specificity	Sensitivity	
**GDF-15** [15, 16]	Diagnosis Prognosis	Blood	On the 1st, 4th and 7th day after ICU admission	Stress response	On the 1st, 4th and 7th day after admission to the ICU	MRC	0.904	1722 pg/mL	0.711	0.940	-
	Predicts	Blood	Enter ICU within 24 h	Stress response	Every day in the ICU	MRC	0.867 (0.824–0.903)	2.16 µg/mL	0.875	0.793	-
**MCP-1** [18]	Diagnosis	Blood	On the 1st, 4th and 7th day after admission to the ICU	Inflammatory process	Every day in the ICU	MRC	**1d**:0.732 (95%CI: 0.629–0.836)	**1d**: 206.3ng/L	**1d**: 0.544	**1d**: 0.871	-
							**4d**:0.865 (95%CI: 0.777–0.953)	**4d**: 410.9ng/L	**4d**: 0.961	**4d**: 0.640	-
							**7d**: 0.891 (95%CI: 0.790–0.986)	**7d**: 239.5ng/L	**7d**: 0.862	**7d**: 0.824	-
**NfH** [20]	Diagnosis	Blood	Every day in the ICU	Neurological injury	After the patient is awake and attentive	MRC	0.850 (95%CI: 0.720–0.970)	17.9 ng/mL	0.810	0.830	-
**N-titin** [24]	Diagnosis	Urine	On the morning after admission (Day 1) and on Days 3, 5, 7	Muscle injury	ICU on days 1 and 10	MRC	0.810 (95%CI: 0.688–0.931)	100 pmol/mg/Cre	0.897	0.619	-
**GLUT-4** [23]	Predicts	Blood	Upon ICU admission	Metabolic pathway	After completely stopping sedative and analgesic drugs for 2 h and being able to cooperate	MRC	0.880 (95%CI: 0.780–0.980)	159.63 ng/L	0.867	0.765	PPV: 0.684 NPV: 0.907
**Myoglobin** [17]	Predicts	Blood	Upon ICU admission	Muscle injury	Every day in the ICU	MRC	0.843 (95%CI: 0.819–0.867)	1362.50 ng/mL	0.823	0.760	-
**NfL** [21]	Predicts	Blood	Enter ICU within 24 h	Neurological injury	Every day in the ICU	MRC	0.735 (95% CI: 0.611–0.837)	103.70 ng/mL	0.667	0.732	-
**Urinary** **titin** [25]	Predicts Prognosis	Urine	Within 12 h of ICU admission and over 24 h on days 2, 3, 5, and 7	Muscle injury	On days 1, 3, 5, and 7 of ICU admission	MRC	**Cumulative level**: 0.780 (95% CI: 0.610–0.950)	**Cumulative level**: 181.5pmol/mg Cre	**Cumulative level**: 0. 770	**Cumulative level**: 0.780	-
							**2d**: 0.750 (95% CI: 0.560–0.940)	**2d**:64.8 pmol/mg Cre	**2d**: 0.810	**2d**: 0.780	-
**NETs marker cfDNA** [22]	Predicts Prognosis	Blood	On the 1st day after admission to the ICU	Inflammatory process	Between the 1st and 12th day of admission to the ICU	MRC	0.786	-	-	-	-
**MiR-181a** [19]	Predicts	Blood	On postoperative days 1, 2, and 7 or at discharge	Inflammatory process	On the 1st and 2nd postoperative days and on day 7 or at discharge from the hospital	B-mode ultrasound imaging	0.600	-	0.910	0.560	PPV: 0.910 NPV: 0.560

ICU: intensive care unit; ICU-AW: intensive care unit-acquired weakness; AUC: area under curve; GDF-15: growth differentiation factor-15; MRC: medical research council scale; MCP-1: monocyte chemoattractant protein-1; miR-181a: microRNA-181a; NfH: neurofilament heavy chain; GLUT-4: glucose transporter type-4; NfL: neurofilament light chain; cfDNA: cell-free DNA; NETs: neutrophil extracellular traps; PPV: Positive Predictive Value; NPV: Negative predictive value; -: Not mention

## Discussion

### Main finding

The present study focuses on systematically reviewing biomarkers associated with the prediction, diagnosis, and prognosis of ICU-AW. Ten biomarkers were identified and categorized into five mechanisms: muscle injury (myoglobin, N-titin and urinary titin), metabolic pathway (GLUT-4), neurological injury (NfL/NfH), stress response (GDF-15), and inflammatory response (MCP-1, NETs marker cfDNA, and miR-181a). Among them, GDF-15 has good performance in prediction, diagnosis, and prognosis. MiR-181a has the lowest predictive performance, and whether it can be widely applied in clinical practice still needs to be extensively verified.

### The potential ICU-AW biomarkers mechanism and performance

#### Muscle injury biomarkers

Muscle injury is characterized by structural disruption of muscle segments, cytoskeletal components, and membrane integrity, coupled with enhanced protein permeability across the compromised membranes [32]. ICU-AW is characterized by disrupted contraction mechanisms, including a significant loss of coarse myosin filament and altered actin-to-myosin ratios [33, 34]. Titin, a large structural protein encoded by the TTN gene, and spaning from the Z-disk to the M-band in muscle cells, supports the contraction of thin actin-containing filaments and myosin-containing thick filaments [35]. As an early myofibrillar protein, its degradation produces urinary N-terminal fragments that indicate early myofibrillar damage and muscle atrophy [36, 37]. In a prospective two-centre study by Nakanishi et al., cumulative urinary titin (AUC 0.780 [95% CI: 0.610–0.950]) slightly outperformed titin measured on day 2 (AUC 0.750 [95% CI: 0.560–0.940]) in predicitng mortality (*p* = 0.02) [25]. Another study of 50 critically ill patients linked mean N-titin/Cre levels with MRC scores (AUC 0.810 [95% CI: 0.688–0.931]), although sensitivity was only 61.9% [24]. While N-titin and urinary titin were promising biomarker for ICU-AW, its reliance on urine limits its applicability in anuric patients [24, 25].

In critically ill patients, a complex cascade of neuromuscular injury, cellular destruction and metabolic abnormalities may lead to myofibril rupture [38, 39], releasing large amounts of myoglobin into circulation [40]. In a study of 211 sepsis patients, serum myoglobin was positively correlated with ICU-AW severity and mortality, with levels > 1362.5 ng/ml indicating an increased risk (AUC 0.843, 95% CI: 0.819–0.867) [17].

Myosin loss was observed in 70% of CIM patients, with the extent of myosin loss showing a modest correlation with mortality, suggesting its potential as a prognostic marker [41]. While serum creatine kinase (CK) has been associated with ICU-AW [42], patients who regained alertness after 7 days of mechanical ventilation exhibited only a slight increase in peak CK levels, with no significant difference between ICU-AW patients and non-ICU-AW patients [43]. However, a recent study found that CK levels were significantly elevated (*p* < 0.05) in cardiogenic shock patients with ICU-AW, suggesting multi-organ involvement [44]. Further research is needed to determine the specificity and validation of these biomarkers for muscle injury.

#### Neurological injury biomarkers

Neurological injury compromises structural integrity and leads to functional abnormalities, including impaired neurological signal transmission [45]. Sepsis can trigger microvascular changes in the Tunica intima, increasing vascular permeability and allowing toxic factors to damage nerve endings, resulting in edema, cell injury, and axonal degeneration [46]. Axonal degeneration is a hallmark of CIP pathology [47]. Neurofilament proteins which provide the elasticity, are intermediate filaments present in the cytoplasm of neurons and are assembled from three specific protein subunit. When axonal injury occurs, these proteins are released into the synaptic cleft, elevating their levels in serum or cerebrospinal fluid [48, 49], and correlating with axonal damage in ICU-AW. Wieske et al. [20] proposed that both CINM and CIP contribute to axonal injury, suggesting that neurofilaments may serve as ICU-AW biomarkers. Due to efficacy analysis limitations, they used ELISA (NfH^SMI35^ antibody) to measure nerve filament levels, and showed that the NfH level in ICU-AW patients was relatively high, with a peak on the 7th day demonstrating good discriminatory ability an optimal threshold of 17.9 ng/ml (AUC 0.850 [95% CI: 0.720–0.970]) [20]. Notably, peak levels were not observed prior to muscle strength assessment, thus supporting the theory that ICU-AW develops after functional impairment and peripheral nerve injury [50]. In a prospective case-control study of coronary care unit patients, the ICU-AW group had significantly higher levels of NfL (405 [IQR 77–835] vs. 27 [IQR 12–90] pg/mL) and mortality [51]. In addition, NfL showed predictive value (cut off 103.70 mg/L, AUC 0.735 [95% CI: 0.611–0.837]) [21].

Recently, biomarkers such as glial fibrillary acidic protein (GFAp) and Tau have garnered attention in CIM patients, as elevated levels of GFAp (*p* = 0.02) and phosphorylated Tau (*p* = 0.04) can identify myelinated axonal lesions in ICU-AW [52]. Although neuromarker progress has been slow due to assay limitations [53], advances in high-sensitivity digital immunoassays—such as the single molecule array platform, which is over 1,200-fold more sensitive than conventional ELISA [54, 55], are expected to recognising much smaller concentrations of substances.

#### Inflammatory biomarkers

As a protective physiological response to infections and tissue injury, the inflammatory process involves various mediators that coordinate the elimination of harmful stimuli and subsequent tissue repair [56]. As the severity of the disease progresses in critically ill patients, inflammatory cytokines produced during the process of important organ damage will further deteriorate muscle function [57]. MCP-1, also known as CCL2, is an inflammation-related biomarker that recruits monocytes/macrophages to injured muscle, facilitating phagocytosis, muscle repair, and insulin-like growth factor 1 release [58, 59]. MCP-1 levels exceeding 239.5 ng/L on day 7 in sepsis patients robustly predict ICU-AW (AUC 0.891 [95% CI: 0.790–0.986]) [18].

NETs contribute to ICU-AW via platelet activation and inflammation, exacerbating lung and muscle injury. Excessive NETs production disrupts the pulmonary microcirculation, impairs alveolar capillary function, and elevates pro-inflammatory cytokines, particularly in COVID-19 and acute respiratory distress syndrome [60, 61], while releasing cfDNA during cell destruction [62]. NETs marker cfDNA further amplifies systemic inflammation and organ dysfunction [63, 64]. Huckriede et al. demonstrated that NETs marker cfDNA levels on ICU admission were associated with ICU-AW (AUC 0.786), and the magnitude of cfDNA change significantly predicts mortality, with an optimal cut-off of -27.22 ng/mL (AUC 0.820, 95% CI 0.631-1.000) [22]. However, the exclusive inclusion of critically ill COVID-19 patients may limit the generalizability of these results.

Muscle specific miR-181a, involved in muscle regeneration and inflammation regulation [65], has been shown to be a useful early biomarker for acute muscle atrophy in critically ill patients [19]. In ICU patients undergoing high-risk elective surgery, higher plasma miR-181a levels on day 2 were highly specificily associated with subsequent muscle atrophy at one week (AUC 0.600) [19]. Although the test exhibits a high positive predictive value (91%) for identifying high-risk patients, its low sensitivity (56%) limits its utility for ruling out acute atrophy.

Cytokines are central to the neuromuscular inflammatory cascade. Witteveen et al. reported that ICU-AW patients exhibited elevated systemic levels of interleukin-6 (IL-6), IL-8, IL-10, and fractalkine (OR 1.35 [95% CI: 1.18–1.55]), with mixed-effects modeling revealing a 1.5- to 2-fold increase in these markers [57]. Elevated levels of tumor necrosis factor -α, IL-1β, and IL-6 in mechanically ventilated patients further increase the risk of ICU-AW and are associated with poor prognosis [66]. However, these cytokine levels may fluctuate during infection, and their specificity for ICU-AW remains unverified. Combining multiple inflammatory markers into a diagnostic panel may prove superior to individual biomarkers [67].

#### Stress response biomarkers

Stress response is a phenomenon caused by the imbalance between the production and accumulation of ROS in cells and tissues and the ability of biological systems to detoxify these reaction products [68]. Sepsis and critical illness lead to increased oxidative stress, reducing ATP formation and causing mitochondrial dysfunction and free radical production [69]. GDF-15, a transforming growth factor-β superfamily member, plays a key role in this stress response [70]. GDF-15 is a powerful diagnostic, predictive, and prognostic biomarker in the ICU environment, and its elevated levels are negatively correlated with muscle mass and strength. In mechanically ventilated ICU patients, plasma GDF-15 levels above 1,722 pg/ml on day 7 providing strong diagnostic performance (AUC 0.904, sensitivity 94.0%) associated with muscle atrophy, and higher levels were associated with significantly lower 90-day survival rate than the low levels group (54.00% vs. 75.56%) [15]. Additionally, early GDF-15 measurements (< 6 h post-admission) predicted ICU-AW with an AUC of 0.867 (cut-off 2.16 µg/L, sensitivity 87.5%) [16].

#### Metabolic pathway biomarkers

The metabolic pathway systematically converts substrates into final products through the coordinated action of enzymes and intermediate metabolites [71]. Muscle protein renewal depends on a balance between synthesis and degradation [72]. Impaired insulin signaling particularly the failure of GLUT-4 translocation reduces protein synthesis and is implicated in critical myopathy [73]. GLUT-4 facilitates rapid glucose uptake, maintains homeostasis, and may participate in metabolic pathways relevant to ICU-AW [23, 73, 74]. In critically ill renal transplant recipients, its expression was significantly lower in the ICU-AW group (137.86 ± 127.87 ng/L) compared to non-ICU-AW (419.15 ± 267.68 ng/L), yielding a negative predictive value (AUC 0.880 [95% CI: 0.780–0.980]) [23]. In constract, AMPK, another glucose metabolism marker, showed no predictive value for ICU-AW. Given the critical role of energy metabolism in ICU-AW pathogenesis, further validation of other AMPK pathway proteins is warranted across diverse patient populations.

### Challenges and recommendations

Currently, modifying ICU-AW clinical diagnostic criteria is neither feasible nor practical. Ideally, diagnosis should begin with patient-initiated assessments like MRC and grip strength testing. For uncooperative patients, ultrasound or imaging can evaluate muscle thickness changes, while electrophysiological exams and muscle/nerve biopsies help differentiate CIP, CIM, and CINM. Noninvasive electrophysiological testing should also be advanced to reduce invasive procedures. However, this multimodal approach may increase costs and time [75].

Biomarkers, as convenient tools, hold potential for large-scale clinical use but remain limited in applicability. We also reviewed ICU-AW diagnostic guidelines and relevant reviews. We only identified one diagnostic guideline and did not mention the use of any specific biomarkers [6]. While some reviews highlight their predictive, diagnostic, and prognostic value, none offer clear clinical recommendations, underscoring persistent challenges [1, 76, 77]. A key issue is the mismatch between MRC scale and biomarker validation results. Most studies use the MRC scale diagnostically, yet its subjectivity and ceiling effect—along with challenges in uncooperative patients—may skew biomarker validation [1]. Heterogeneous ICU populations (e.g., age, comorbidities) impact validation [78], while disease mechanisms or treatments may alter biomarker levels [79]. Many studies rely on single time-point measurements, hindering dynamic monitoring, and most lack external validation or covariate analysis [52, 66]. Only a few studies reported additional parameters, such as positive and negative predictive values.

Future work should focus on discovering novel biomarkers and optimizing existing ones, enhancing reliability through stratified analysis and multicenter validation [80]. Developing biomarker panels for dynamic monitoring and multi-pathway integration could improve diagnostic accuracy [81]. Finally, combining multi-omics with artificial intelligence and machine learning to analyze large datasets may address current limitations [82–84].

### Strengths and limitations

The primary strength of this review is that it represents the first extensive, standardized search of ICU-AW biomarker studies, providing a comprehensive profile of their mechanisms, performance, and clinical applicability. We anticipate that these biomarkers may serve as valuable clinical tools while potentially accelerating the identification of novel biomarkers through their mechanistic insights. However, several limitations exist. (1) We excluded animal studies, which, although informative, pose challenges in critical evaluation and clinical relevance. (2) We included only performance-validated biomarkers, excluded studies lacking validation results. (3) The heterogeneity among the included studies precluded quantitative analysis, although we provided a comprehensive qualitative synthesis to mitigate potential biases. (4) Restricting our search to English and Chinese publications may have introduced language bias.

## Conclusion

The emergence of biomarkers offers promising potential for advancing ICU-AW research. However, despite the availability of ten biomarkers, their clinical utility remains limited, highlighting the need for large-scale prospective validation to strengthen the evidence for clinical practice. Given the complex pathophysiology and the impact of interventions on ICU-AW, evaluating biomarker panels and conducting stratified analyses are crucial for enhancing detection and monitoring in critically ill patients, thereby addressing a critical gap in clinical care.

## Electronic supplementary material

Below is the link to the electronic supplementary material.

Supplementary Material 1

## Data Availability

The datasets used and/or analyzed during the current study are available from the corresponding author on reasonable request.

## Abbreviations

AUC: Area under curve
cfDNA: Cell free DNA
CI: Confidence interval
CIM: Critical illness myopathy
CINM: Critical illness neuromyopathy
CIP: Critical illness polyneuropathy
CK: Creatine kinase
ELISA: Enzyme linked immunosorbent assay
GDF-15: Growth differentiation factor-15
GFAp: Glial fibrillary acidic protein
GLUT-4: Glucose transporter type-4
ICU: Intensive care unit
ICU-AW: Intensive care unit-acquired weakness
IL-6: Interleukin-6
MCP-1: Monocyte chemoattractant protein-1
miR-181a: microRNA-181a
MRC: Medical research council
NETs: Neutrophil extracellular traps
NfH: Neurofilament heavy chain
NfL: Neurofilament light chain
NOS: Newcastle-ottawa scale
qPCR: Quantitative-real time polymerase chain reaction
QUADAS-2: Quality assessment of diagnostic accuracy studies-2
UK: The United Kingdom
