# Supplementary material for: Biomarkers for intensive care unit-acquired weakness: a systematic review for prediction, diagnosis and prognosis
Source: Ann Intensive Care. 2025 Jul 2;15:86. doi: 10.1186/s13613-025-01500-9 (PMC12222590; doi:10.1186/s13613-025-01500-9)
Supplement: Supplementary file 1 — Supplementary Material 1 [file 13613_2025_1500_MOESM1_ESM.docx]

Table S1 The search strategy and results for 8 databases

| CNKI | | |
| --- | --- | --- |
| 23/1/2025 | | |
| #1 | SU=危重病性多发性神经病 + 危重病性多发性神经肌病 + 危重病性肌病 + 危重病性疾病性多发性神经病 + 危重病性疾病性多发性神经肌病 + 危重病性疾病性肌病 + 危重症多发性肌病 + 危重症多发性神经病危重症多发性神经肌病 + 危重症肌病 +危重症神经肌病 + 多发性神经危重疾病 + 重症神经肌肉异常 + CIM + CINM + CINMA + CIP + CIPNM + ICUAW + ICU获得性肌无力 + ICU获得性麻痹 + ICU获得性衰弱 + ICU获得性虚弱 + 重症监护病房获得性肌无力 + 重症监护室获得性肌无力 + ICU后综合征 + PICS + 重症监护室后综合征 | 1.56万 |
| #2 | SU=ICU + 重症监护病房 + 重症监护 + 重症监护室 + 重症 + 危重 + 危重症 + 脓毒症 + 脓毒症休克 + 脓毒血症 | 24.98万 |
| #3 | SU=获得性肌病 + 获得性肌无力 + 获得性麻痹 + 获得性神经肌肉疾病 + 获得性神经肌肉紊乱 + 获得性衰弱 + 相关性肌无力 + 相关性衰弱 + 相关性虚弱 + 获得性虚弱 + 四肢轻瘫 + 获得性瘫痪 + 肌疾病 + 虚弱 + 衰弱 + 神经肌肉障碍 + 肌病 + 膈肌萎缩 + 呼吸肌萎缩 + 呼吸肌无力 + 膈肌无力 | 2.6万 |
| #4 | #2 AND #3 | 1006 |
| #5 | #1 OR #4 | 1.61万 |
| #6 | SU=生物标志物+生物标记物+诊断+预后+预测+死亡+生存+存活 | 290.28万 |
| #7 | #5 AND #6 | 817 |
| Wanfang Database | | |
| 23/1/2025 | | |
| #1 | 题名或关键词:(危重病性多发性神经病 or 危重病性多发性神经肌病 or 危重病性肌病 or 危重病性疾病性多发性神经病 or 危重病性疾病性多发性神经肌病 or 危重病性疾病性肌病 or 危重症多发性肌病 or 危重症多发性神经病危重症多发性神经肌病 or 危重症肌病 or危重症神经肌病 or 多发性神经危重疾病 or 重症神经肌肉异常 or CIM or CINM or CINMA or CIP or CIPNM or ICUAW or ICU获得性肌无力 or ICU获得性麻痹 or ICU获得性衰弱 or ICU获得性虚弱 or 重症监护病房获得性肌无力 or 重症监护室获得性肌无力 or ICU后综合征 or PICS or 重症监护室后综合征) | 12,497 |
| #2 | 题名或关键词:(ICU or 重症监护病房 or 重症监护 or 重症监护室 or 重症 or 危重 or 危重症 or 脓毒症 or 脓毒症休克 or 脓毒血症) and 题名或关键词:(获得性肌病 or 获得性肌无力 or 获得性麻痹 or 获得性神经肌肉疾病 or 获得性神经肌肉紊乱 or 获得性衰弱 or 相关性肌无力 or 相关性衰弱 or 相关性虚弱 or 获得性虚弱 or 四肢轻瘫 or 获得性瘫痪 or 肌疾病 or 虚弱 or 衰弱 or 神经肌肉障碍 or 肌病 or 膈肌萎缩 or 呼吸肌萎缩 or 呼吸肌无力 or 膈肌无力) | 1,826 |
| #3 | #1 or #2 | 13,619 |
| #4 | 题名或关键词:(生物标志物 or 生物标记物 or 诊断 or 预后 or 预测 or 死亡 or 生存 or 存活） | 2,503,853 |
| #5 | #3 and #4 | 513 |
| China Biomedical Literature Database (SinoMed) | | |
| 23/1/2025 | | |
| #1 | "危重病性多发性神经病"[标题:智能] OR "危重病性多发性神经肌病"[标题:智能] OR "危重病性肌病"[标题:智能] OR "危重病性疾病性多发性神经病"[标题:智能] OR "危重病性疾病性"[标题:智能] OR "多发性神经肌病"[标题:智能] OR "危重病性疾病性肌病"[标题:智能] OR "危重症多发性肌病"[标题:智能] OR "危重症多发性神经病危重症多发性神经肌病"[标题:智能] OR "危重症肌病"[标题:智能] OR "危重症神经肌病"[标题:智能] OR "多发性神经危重疾病"[标题:智能] OR "重症神经肌肉异常"[标题:智能] OR "CIM"[标题:智能] OR "CINM"[标题:智能] OR "CINMA"[标题:智能] OR "CIP"[标题:智能] OR "CIPNM"[标题:智能] OR "ICU-AW"[标题:智能] OR "ICU获得性肌无力"[标题:智能] OR "ICU获得性麻痹"[标题:智能] OR "ICU获得性衰弱"[标题:智能] OR "ICU获得性虚弱"[标题:智能] OR "重症监护病房获得性肌无力"[标题:智能] OR "重症监护室获得性肌无力"[标题:智能] OR "ICU后综合征"[标题:智能] OR "PICS"[标题:智能] OR "重症监护室后综合征"[标题:智能] | 1,060 |
| #2 | "ICU"[中文标题:智能] OR "重症监护病房"[中文标题:智能] OR "重症监护"[中文标题:智能] OR "重症监护室"[中文标题:智能] OR "重症"[中文标题:智能] OR "危重"[中文标题:智能] OR "危重症"[中文标题:智能] OR "脓毒症"[中文标题:智能] OR "脓毒症休克"[中文标题:智能] OR "脓毒血症"[中文标题:智能] | [174,243](javascript:void(0);) |
| #3 | ( "获得性肌病"[标题:智能] OR "获得性肌无力"[标题:智能] OR "获得性麻痹"[标题:智能] OR "获得性神经肌肉疾病"[标题:智能] OR "获得性神经肌肉紊乱"[标题:智能] OR "获得性衰弱"[标题:智能] OR "相关性肌无力"[标题:智能] OR "相关性衰弱获得性"[标题:智能] OR "四肢轻瘫"[标题:智能] OR "获得性瘫痪"[标题:智能] OR "神经肌肉障碍"[标题:智能] OR "膈肌萎缩"[标题:智能] OR "呼吸肌萎缩"[标题:智能] OR "呼吸肌无力"[标题:智能] OR "膈肌无力"[标题:智能] OR "获得性虚弱"[标题:智能] OR "虚弱"[标题:智能] OR "衰弱"[标题:智能] OR "相关性虚弱"[标题:智能]) | 5829 |
| #4 | #2 AND #3 | 497 |
| #5 | #1 OR #4 | 1,215 |
| #6 | 生物标志物 OR 生物标记物 OR 诊断 OR 预后 OR 预测 OR 死亡 OR 生存 or 存活 | [747,300](javascript:void(0);) |
| #7 | #5 AND #6 | 106 |
| China Science and Technology Journal Database (VIP Database) | | |
| 23/1/2025 | | |
| #1 | (M=(生物标志物 or 生物标记物 or 诊断 or 预后 or 预测)) and ((M=(危重病性多发性神经病 or 危重病性多发性神经肌病 or 危重病性肌病 or 危重病性疾病性多发性神经病 or 危重病性疾病性多发性神经肌病 or 危重病性疾病性肌病 or 危重症多发性肌病 or 危重症多发性神经病危重症多发性神经肌病 or 危重症肌病 or 危重症神经肌病 or 多发性神经危重疾病 or 重症神经肌肉异常 or CIM or CINM or CINMA or CIP or CIPNM or ICUAW or ICU获得性肌无力 or ICU获得性麻痹 or ICU获得性衰弱 or ICU获得性虚弱 or 重症监护病房获得性肌无力 or 重症监护室获得性肌无力) or M=((ICU or 重症监护病房 or 重症监护 or 重症监护室 or 重症 or 危重 or 危重症 or 脓毒症 or 脓毒症休克 or 脓毒血症) and (获得性肌病 or 获得性肌无力 or 获得性麻痹 or 获得性神经肌肉疾病 or 获得性神经肌肉紊乱 or 获得性衰弱 or 相关性肌无力 or 相关性衰弱获得性 or 四肢轻瘫 or 获得性瘫痪 or 肌疾病 or 神经肌肉障碍 or 肌病 or 膈肌萎缩 or 呼吸肌萎缩 or 呼吸肌无力 or 膈肌无力)))) not (R=动物 or c2c12 or 兔 or 鼠 or 猪 or 鱼 or 细胞) | 398 |
| Pubmed | | |
| 23/1/2025 | | |
| #1 | CIM[Title/Abstract] OR CINM[Title/Abstract] OR CINMA[Title/Abstract] OR CIP[Title/Abstract] OR CIPNM[Title/Abstract] OR Critical illness myopathy[Title/Abstract] OR Critical illness myopathy[Title/Abstract] OR Critical illness neuromuscular abnormality[Title/Abstract] OR Critical illness neuromyopathy[Title/Abstract] OR Critical illness polyneuromypathy[Title/Abstract] OR Critical illness polyneuropathies[Title/Abstract] OR Critical illness polyneuropathy[Title/Abstract] OR Critical illness polyneuropathy[Title/Abstract] OR ICU acquired weakness[Title/Abstract] OR ICUAP[Title/Abstract] OR ICUAW[Title/Abstract] OR Intensive care unit acquired paresis[Title/Abstract] OR Intensive care unit acquired weakness[Title/Abstract] OR septic myopathy[Title/Abstract] OR septic weakness[Title/Abstract] OR saw[Title/Abstract] OR sepsis acquired weakness[Title/Abstract] OR post-intensive care syndrome[Title/Abstract] OR post-ICU syndrome[Title/Abstract] OR PICS[Title/Abstract] | 40,906 |
| #2 | (ICU[Title/Abstract] OR Critical ill[Title/Abstract] OR Critically Illness[Title/Abstract] OR Critical Care[Title/Abstract] OR sepsis[Title/Abstract]) AND (acquired neuromuscular disease[Title/Abstract] OR acquired neuromuscular disorder[Title/Abstract] OR acquired neuromuscular dysfunction[Title/Abstract] OR acquired polyneuropathies[Title/Abstract] OR acquired polyneuropathy[Title/Abstract] OR acquired quadriplegia[Title/Abstract] OR Polyneuropathy[Title/Abstract] OR motor polyneuropathies[Title/Abstract] OR Polyneuropathy[Title/Abstract] OR muscle atrophy[Title/Abstract] OR diaphragm weakness[Title/Abstract] OR [frailty](file:///D:/%E6%A1%8C%E9%9D%A2%E8%BD%AF%E4%BB%B6/baidu-translate-client/resources/app.asar/app.html#/#)[Title/Abstract]) | 1,724 |
| #3 | ("Biomarkers"[Mesh]) OR (biomarkers[Title/Abstract] OR biochemical marker[Title/Abstract] OR biomarker[Title/Abstract] OR biological marker[Title/Abstract] OR diagnose[Title/Abstract] OR prognosis[Title/Abstract] OR prediction[Title/Abstract] OR prognosis[Title/Abstract] OR mortality[Title/Abstract] OR survival[Title/Abstract]) | 3,932,628 |
| #4 | #1 OR #2 | 42,226 |
| #5 | #3 AND #4 | 5,077 |
| #6 | #5 NOT ((animal[Title/Abstract] OR mice[Title/Abstract] OR rat[Title/Abstract] OR mouse[Title/Abstract] OR cell[Title/Abstract] OR cellular[Title/Abstract] OR dog[Title/Abstract] OR fish[Title/Abstract] OR C2C12[Title/Abstract] OR rabbit[Title/Abstract] OR pig[Title/Abstract])) | 4,082 |
| #7 | #6  Filters: Humans | 2,945 |
| Embase | | |
| 23/1/2025 | | |
| #1 | biomarkers:ti OR 'biochemical marker':ti OR biomarker:ti OR 'biological marker':ti OR diagnose:ti OR prediction:ti OR prognosis:ti OR mortality:ti OR survival:ti | 946,814 |
| #2 | icu:ab OR 'critical ill':ab OR 'critically illness':ab OR 'critical care':ab OR sepsis:ab | 390,288 |
| #3 | 'acquired neuromuscular disease':ab OR 'acquired neuromuscular disorder':ab OR 'acquired neuromuscular dysfunction':ab OR 'acquired polyneuropathies':ab OR 'acquired polyneuropathy':ab OR 'cquired quadriplegia':ab OR 'motor polyneuropathies':ab OR polyneuropathy:ab OR 'muscle atrophy':ab OR 'diaphragm weakness':ab OR frailty:ab | 80,734 |
| #4 | cim:ab OR cinm:ab OR cinma:ab OR cip:ab OR cipnm:ab OR 'critical illness myopathy':ab OR 'critical illness neuromuscular abnormality':ab OR 'critical illness neuromyopathy':ab OR 'critical illness polyneuromypathy':ab OR 'critical illness polyneuropathies':ab OR 'critical illness polyneuropathy':ab OR 'icu acquired weakness':ab OR icuap:ab OR icuaw:ab OR 'intensive care unit acquired paresis':ab OR 'intensive care unit acquired weakness':ab OR 'septic myopathy':ab OR 'septic weakness':ab OR 'sepsis acquired weakness':ab | 12,127 |
| #5 | #2 AND #3 | 2,989 |
| #6 | #4 OR #5 | 14,599 |
| #7 | #1 AND #6 | 558 |
| Cochrone library | | |
| 23/1/2025 | | |
| #1 | (ICU or Critical ill or Critically Illness or Critical Care):kw AND (acquired neuromuscular disease or acquired neuromuscular disorder or acquired neuromuscular dysfunction or acquired polyneuropathies or acquired polyneuropathy or acquired quadriplegia or Polyneuropathy or motor polyneuropathies or Polyneuropathy or muscle atrophy or diaphragm weakness or [frailty](file:///D:/%E6%A1%8C%E9%9D%A2%E8%BD%AF%E4%BB%B6/baidu-translate-client/resources/app.asar/app.html#/#)):kw | 91 |
| #2 | cim:kw OR cinm:kw OR cinma:kw OR cip:kw OR cipnm:kw OR 'critical illness myopathy':kw OR 'critical illness neuromuscular abnormality':kw OR 'critical illness neuromyopathy':kw OR 'critical illness polyneuromypathy':kw OR 'critical illness polyneuropathies':kw OR 'critical illness polyneuropathy':kw OR 'critical illness polyneuropathy':kw OR 'icu acquired weakness':kw OR icuap:kw OR icuaw:kw OR 'intensive care unit acquired paresis':kw OR 'intensive care unit acquired weakness':kw OR 'septic myopathy':kw OR 'septic weakness':kw OR saw:kw OR 'sepsis acquired weakness':kw OR 'post-intensive care syndrome':kw OR 'post-icu syndrome':kw OR pics:kw | 2,508 |
| #3 | (biomarkers or biochemical marker or biomarker or biological marker or diagnose or prognosis or prediction or prognosis or mortality or survival):ab | 253,388 |
| #4 | #1 OR #2 | 2,574 |
| #5 | #3 AND #4 | 565 |
| Web of Science | | |
| 23/1/2025 | | |
| #1 | TI=(CIM or CINM or CINMA or CIP or CIPNM or Critical illness myopathy or Critical illness myopathy or Critical illness neuromuscular abnormality or Critical illness neuromyopathy or Critical illness polyneuromypathy or Critical illness polyneuropathies or Critical illness polyneuropathy or Critical illness polyneuropathy ICU acquired weakness or ICUAP or ICUAW or Intensive care unit acquired paresis or Intensive care unit acquired weakness or septic myopathy or septic weakness or saw or sepsis acquired weakness or post-intensive care syndrome or post-ICU syndrome or PICS) | 23,653 |
| #2 | TI=(ICU or Critical ill or Critically Illness or Critical Care) | 21,720 |
| #3 | TI=(acquired neuromuscular disease or acquired neuromuscular disorder or acquired neuromuscular dysfunction or acquired polyneuropathies or acquired polyneuropathy or acquired quadriplegia or Polyneuropathy or motor polyneuropathies or Polyneuropathy or muscle atrophy or diaphragm weakness or [frailty](file:///D:/%E6%A1%8C%E9%9D%A2%E8%BD%AF%E4%BB%B6/baidu-translate-client/resources/app.asar/app.html#/#)) | 22,120 |
| #4 | #2 AND #3 | 93 |
| #5 | #1 OR #4 | 23,739 |
| #6 | TI=(biomarkers or biochemical marker or biomarker or biological marker or diagnose or prognosis or prediction or prognosis or mortality or survival) | 687,104 |
| #7 | #5 AND #6 | 534 |

**Table S2 Quality assessment of cohort studies included in the systematic review according to Newcastle-Ottawa scale**

| Cohort studies | | | | | | | | | | | |
| --- | --- | --- | --- | --- | --- | --- | --- | --- | --- | --- | --- |
| Study | Reviewers | Selection | | | | Comparability | Outcome/Exposure | | | Score | Average score |
|  |  | sample representativeness | Selection of the Non-Exposed Cohort | Ascertainment of Exposure | Demonstration That Outcome of Interest Was Not Present at Start of Study | Comparability of Cohorts on the Basis of the Design or Analysis | Assessment of Outcome | Was Follow-Up Long Enough for Outcomes to Occur | Adequacy of Follow Up of Cohorts |  |  |
| Wang 2022 [1] | ① | 1 | 1 | 1 | 1 | 2 | 0 | 1 | 1 | 8 | 7.5 |
|  | ② | 1 | 1 | 0 | 1 | 2 | 0 | 1 | 1 | 7 |  |
| Ding 2022 [2] | ① | 1 | 1 | 1 | 1 | 2 | 0 | 1 | 1 | 8 | 8 |
|  | ② | 1 | 1 | 0 | 1 | 2 | 1 | 1 | 1 | 8 |  |
| Zhao 2023 [3] | ① | 1 | 1 | 1 | 0 | 0 | 1 | 1 | 1 | 6 | 6 |
|  | ② | 1 | 1 | 0 | 1 | 0 | 1 | 1 | 1 | 6 |  |
| Wang 2024 [4] | ① | 1 | 1 | 1 | 1 | 2 | 0 | 1 | 1 | 8 | 7.5 |
|  | ② | 1 | 1 | 0 | 1 | 2 | 0 | 1 | 1 | 7 |  |
| Bloch 2015 [5] | ① | 1 | 1 | 1 | 1 | 2 | 0 | 1 | 1 | 8 | 7.5 |
|  | ② | 1 | 1 | 0 | 1 | 2 | 0 | 1 | 1 | 7 |  |
| Nakanishi 2020 [6] | ① | 1 | 1 | 1 | 0 | 2 | 1 | 0 | 0 | 6 | 6 |
|  | ② | 1 | 1 | 0 | 1 | 2 | 1 | 0 | 0 | 6 |  |
| Nakano 2021[7] | ① | 1 | 1 | 1 | 0 | 2 | 0 | 0 | 1 | 6 | 6.5 |
|  | ② | 1 | 1 | 0 | 1 | 2 | 1 | 0 | 1 | 7 |  |
| Xie 2020 [8] | ① | 1 | 1 | 1 | 0 | 2 | 0 | 1 | 1 | 7 | 7.5 |
|  | ② | 1 | 1 | 0 | 1 | 2 | 1 | 1 | 1 | 8 |  |
| Wieske 2014 [9] | ① | 1 | 1 | 0 | 1 | 2 | 1 | 0 | 1 | 7 | 7 |
|  | ② | 1 | 1 | 0 | 1 | 2 | 1 | 0 | 1 | 7 |  |
| Guo 2023 [10] | ① | 1 | 1 | 1 | 1 | 2 | 1 | 0 | 0 | 7 | 7 |
|  | ② | 1 | 1 | 1 | 1 | 2 | 1 | 0 | 0 | 7 |  |
| Huckriede 2021 [11] | ① | 1 | 1 | 1 | 1 | 2 | 0 | 0 | 0 | 6 | 6.5 |
|  | ② | 1 | 1 | 1 | 1 | 2 | 1 | 0 | 0 | 7 |  |

**Table S3** **QUADAS-2 scale for evaluation of diagnostic studies**

| **Domain** | **Topic** | **Guided questions** | **Detailed Scoring of Literature** | | | | | | | | |
| --- | --- | --- | --- | --- | --- | --- | --- | --- | --- | --- | --- |
|  |  |  | **Xie 2020 [8]** | | **Ding 2022 [2]** | | **Wieske 2014 [9]** | | **Nakano 2021 [7]** | | |
|  |  |  | Reviewer ① | Reviewer ② | Reviewer ① | Reviewer ② | Reviewer ① | Reviewer ② | | Reviewer ① | Reviewer ② |
| **Patient Selection** | Risk of bias | **Could the selection of patients have introduced bias?**  Was a consecutive or random sample of patients enrolled?Was a case-control design avoided?  Did the study avoid inappropriate exclusions? | Low risk | Low risk | Low risk | Low risk | Low risk | Low risk | | Low risk | Low risk |
|  | Applicability | Are there concerns that the included patients and setting do not match the review question? | Low concern | Low concern | Low concern | Low concern | Low concern | Low concern | | Low concern | Low concern |
| **Index test** | Risk of bias | **Could the conduct or interpretation of the index test have introduced bias?**  Were the index test results interpreted without knowledge of the results of the reference standard?  If a threshold was used, was it pre-specified? | Low risk | Low risk | Unclear risk | Unclear risk | Low risk | Low risk | | Low risk | Low risk |
|  | Applicability | Are there concerns that the index test, its conduct, or interpretation differ from the review question? | Low concern | Low concern | Low concern | Low concern | Low concern | Low concern | | Low concern | Low concern |
| **Reference Standard** | Risk of bias | **Could the reference standard, its conduct, or its interpretation have introduced bias?**  Is the reference standards likely to correctly classify the target condition?  Were the reference standard results interpreted without knowledge of the results of the index tests? | Low risk | Unclear risk | Low risk | Low risk | Low risk | Low risk | | Unclear risk | Unclear risk |
|  | Applicability | Are there concerns that the target condition as defined by the reference standard does not match the question? | Low concern | Low concern | Low concern | Low concern | Low concern | Low concern | | Unclear concern | Unclear concern |
| **Flow and Timing** | Risk of bias | **Could the patient flow have introduced bias?**  Was there an appropriate interval between index test and reference standard?  Did all patients receive the same reference standard?  Were all patients included in the analysis? | Unclear risk | Low risk | Unclear risk | Unclear risk | Low risk | Unclear risk | | Unclear risk | Unclear risk |

1. Wang Y, Ma J, Zhang R, Li L. Predictive value of glucose transplantation recipients transporter type 4 for intensive care unit acquired weakness in liver. Chin J Organ fransplant. 2022;43(9):525-9.

2. Ding M, Ren S, Dong X, Wang X, Zhao X, Qin B. Diagnostic accuracy of musele ultrasound and plasma monocyte chemoattractant protein-1 for ICU-acquired weakness in patients with sepsis. Chin Crit Care Med 2022;34(1):12-7.

3. Zhao X, Ding M, Shao H, Qin B. Value of plasma neurofilament light chain to the prediction of ICU-acquired weakness in patients with sepsis. J Chin Pract Diagn Ther 2023;37(03):304-7.

4. Wang L, Long D. Correlation Between Early Serum Myoglobin Levels and the Incidence and Prognosis of Intensive Care Unit-Acquired Weakness (ICU-AW) in Septic Shock Patients: A Comparative Study. An Acad Bras Cienc. 2024;96(2):e20231164.

5. Bloch SA, Donaldson AV, Lewis A, Banya WA, Polkey MI, Griffiths MJ, et al. MiR-181a: a potential biomarker of acute muscle wasting following elective high-risk cardiothoracic surgery. Crit Care. 2015;19(1):147.

6. Nakanishi N, Tsutsumi R, Hara K, Matsuo M, Sakaue H, Oto J. Urinary Titin N-Fragment as a Biomarker of Muscle Atrophy, Intensive Care Unit-Acquired Weakness, and Possible Application for Post-Intensive Care Syndrome. J Clin Med. 2021;10(4).

7. Nakano H, Hashimoto H, Mochizuki M, Naraba H, Takahashi Y, Sonoo T, et al. Urine Titin N-Fragment as a Biomarker of Muscle Injury for Critical Illness Myopathy. Am J Respir Crit Care Med. 2021;203(4):515-8.

8. Xie Y, Liu S, Zheng H, Cao L, Liu K, Li X. Utility of Plasma GDF-15 for Diagnosis and Prognosis Assessment of ICU-Acquired Weakness in Mechanically Ventilated Patients: Prospective Observational Study. Biomed Res Int. 2020;2020:3630568.

9. Wieske L, Witteveen E, Petzold A, Verhamme C, Schultz MJ, van Schaik IN, et al. Neurofilaments as a plasma biomarker for ICU-acquired weakness: an observational pilot study. Crit Care. 2014;18(1):R18.

10. Guo X, Wu X, Xin B. Correlation between serum growth differentiation factor-15 level and intensive care unit-acquired weakness in patients with sepsis requiring mechanical ventilation. J Anhui Med. 2023;44(12):1426-31.

11. Huckriede J, Anderberg SB, Morales A, de Vries F, Hultström M, Bergqvist A, et al. Evolution of NETosis markers and DAMPs have prognostic value in critically ill COVID-19 patients. Sci Rep. 2021;11(1):15701.
